# Partial anomalous pulmonary venous drainage in patients presenting with suspected pulmonary hypertension: A series of 90 patients from the ASPIRE registry

**DOI:** 10.1111/resp.13815

**Published:** 2020-04-06

**Authors:** Robert A. Lewis, Catherine G. Billings, Aidan Bolger, Sarah Bowater, Athanasios Charalampopoulos, Paul Clift, Charlie A. Elliot, Kate English, Neil Hamilton, Catherine Hill, Judith Hurdman, Petra J. Jenkins, Christopher Johns, Simon MacDonald, James Oliver, Vasilios Papaioannou, Smitha Rajaram, Ian Sabroe, Andy J. Swift, A.A. Roger Thompson, David G. Kiely, Robin Condliffe

**Affiliations:** ^1^ Sheffield Pulmonary Vascular Disease Unit Royal Hallamshire Hospital Sheffield UK; ^2^ Department of Adult Congenital Cardiology Glenfield Hospital Leicester UK; ^3^ Department of Adult Congenital Cardiology Queen Elizabeth Hospital Birmingham UK; ^4^ Department of Adult Congenital Cardiology Leeds General Infirmary Leeds UK; ^5^ Department of Academic Radiology University of Sheffield Sheffield UK; ^6^ Department of Adult Congenital Cardiology Manchester Royal Infirmary Manchester UK; ^7^ Department of Infection Immunity and Cardiovascular Disease, University of Sheffield Sheffield UK

**Keywords:** anomalous pulmonary venous drainage, atrial septal defect, pulmonary hypertension, sinus venosus

## Abstract

**Background and objective:**

There are limited data regarding patients with PAPVD with suspected and diagnosed PH.

**Methods:**

Patients with PAPVD presenting to a large PH referral centre during 2007–2017 were identified from the ASPIRE registry.

**Results:**

Ninety patients with PAPVD were identified; this was newly diagnosed at our unit in 71 patients (78%), despite 69% of these having previously undergone CT. Sixty‐seven percent had a single right superior and 23% a single left superior anomalous vein. Patients with an SV‐ASD had a significantly larger RV area, pulmonary artery and L‐R shunt and a higher % predicted DL_CO_ (all *P* < 0.05). Sixty‐five patients were diagnosed with PH (defined as mPAP ≥ 25 mm Hg), which was post‐capillary in 24 (37%). No additional causes of PH were identified in 28 patients; 17 of these (26% of those patients with PH) had a PVR > 3 WU. Seven of these patients had isolated PAPVD, five of whom (8% of those patients with PH) had anomalous drainage of a single pulmonary vein.

**Conclusion:**

Undiagnosed PAPVD with or without ASD may be present in patients with suspected PH; cross‐sectional imaging should therefore be specifically assessed whenever this diagnosis is considered. Radiological and physiological markers of L‐R shunt are higher in patients with an associated SV‐ASD. Although many patients with PAPVD and PH may have other potential causes of PH, a proportion of patients diagnosed with PAH have isolated PAPVD in the absence of other causative conditions.

## Abstract 2

PAPVD was frequently missed in patients presenting with suspected PH. L‐R shunt was higher in patients with associated ASD. Although patients may have other potential causes of PH, some patients with PAH have isolated PAPVD without other causative conditions.

**See related** Editorial

## INTRODUCTION

Anomalous pulmonary venous drainage (APVD) describes a pattern where one of more of the pulmonary veins do not drain into the left atrium but instead are connected to the right atrium, superior or inferior vena cava, azygous vein, coronary sinus or brachiocephalic vein. The prevalence of APVD in adults was found to be 0.1% in a large study involving 45 538 consecutive thoracic computed tomography (CT) scans.^1^

Total APVD presents early in life with neonatal distress and circulatory compromise. Partial APVD (PAPVD) may present later in life due to symptoms related to a volume‐loaded right ventricle (as a result of left‐to‐right (L‐R) shunting) or subsequent development of pulmonary arterial (PA) hypertension (PAH).^2^, ^3^ Alternatively, patients may remain asymptomatic and PAPVD be diagnosed incidentally. PAPVD is commonly associated with other congenital heart defects, particularly sinus venosus atrial septal defects (SV‐ASD).^4^, ^5^, ^6^, ^7^ Once diagnosed, management of patients with PAPVD may involve surgical correction, may be conservative or may involve medical therapy.^2^

Due to right ventricular (RV) dilatation resulting from L‐R shunting and/or the development of pulmonary hypertension (PH), patients with PAPVD may present to PH referral centres but there are few published data regarding patients with suspected or proven PH and coexisting APVD. We therefore performed a study to assess patients with PAPVD presenting to a large PH referral centre over a 10‐year period.

## METHODS

Records for all patients seen at a large PH referral centre between 2007 and 2017 were assessed for evidence of APVD. Approval by the relevant ethics committee was sought and gained (STH14169, NHS Research Ethics Committee 16/YH/0352), and written consent was waived.

Hospital databases, including the ASPIRE (Assessing the Spectrum of Pulmonary hypertension Identified at a REferral centre) registry and electronic medical records, were interrogated for keywords including ‘anomalous’, ‘pulmonary venous drainage’, ‘pulmonary venous return’, ‘scimitar’ and ‘sinus venosus’. Patients who had undergone corrective surgery for PAPVD prior to being seen were excluded, as were those whose cases had been referred or discussed, but not formally seen at our centre. Data regarding associated structural abnormalities, comorbidities, pulmonary haemodynamics and mortality status at the census date of 31 May 2017 were collected. Pulmonary venous anatomy had been assessed on CT imaging (contrast‐enhanced in 88 patients). Cardiac chamber area, PA and aortic diameter were subsequently measured by one author (R.A.L.), blinded to clinical details. Cardiac magnetic resonance (CMR) imaging had been performed in a proportion of patients; pulmonary to systemic shunt assessments (Qp:Qs) derived from phase‐contrast flow measurements were retrieved. Spirometry was available for 97% and percent‐predicted diffusing capacity for carbon monoxide (DL_CO_ %pred) for 89% of patients. At cardiac catheterization, cardiac output was measured by thermodilution. PH was defined as mean PA pressure (mPAP) ≥ 25 mm Hg, in keeping with the definition at the time of study enrolment.

Patients with uncorrected PAPVD who had undergone corrective surgery for an ASD were included in the study but were excluded from comparison against patients with isolated PAPVD. Regional adult congenital heart disease (ACHD) centres were contacted to establish whether patients seen at our centre had subsequently received any surgical intervention for PAPVD or ASD.

Statistical analysis was performed using SPSS v25 (IBM, New York, NY, USA) and GraphPad Prism v8 (San Diego, CA, USA). Continuous data were presented with mean ± SD. Comparison between groups was performed using the unpaired t‐test and response to treatment using the paired t‐test. A *P*‐value of <0.05 was considered significant.

## RESULTS

### Demographics

**Figure 1:**
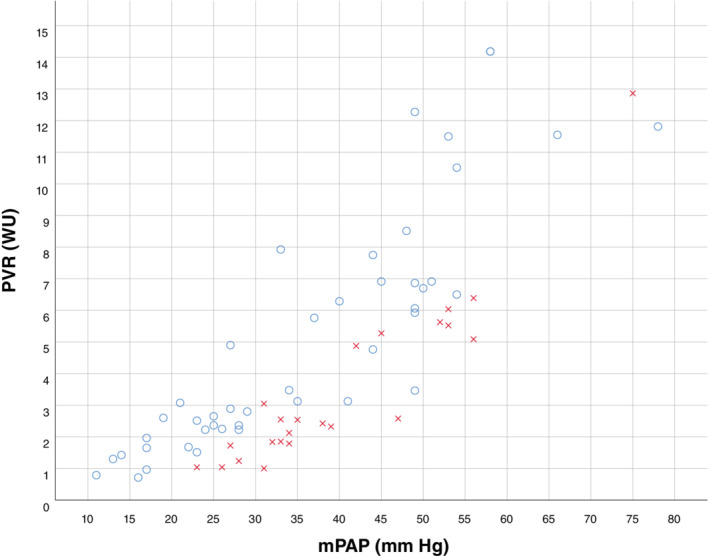
Scatter plot demonstrating haemodynamic parameters for all patients with PAPVD, categorized by PAWP ≤15 mm Hg (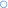) and >15 mm Hg (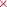). mPAP, mean PA pressure; PA, pulmonary arterial; PAPVD, partial anomalous pulmonary venous drainage; PAWP, PA wedge pressure; PVR, pulmonary vascular resistance.

Ninety patients with PAPVD were identified from our departmental databases. Demographics and haemodynamic and radiological data are displayed in Table 1. Hemodynamic data are displayed visually in Figure 1. The majority of patients were female (70%) with a mean age of 60 years. During the course of the study, 4240 patients were assessed and assigned a specific PH diagnosis at our unit, resulting in an estimated prevalence of 2.1% in this population.

**Table 1:** Baseline demographics

Age (years)	60.4 ± 15.2
Gender (% female)	70
WHO functional class I/II/III/IV (%)	0/24/68/8
Lung function	
FEV_1_ (%pred)	70.4 ± 20.5
FVC (%pred)	85.0 ± 22.4
FEV_1_/FVC (%)	68.3 ± 11.4
DL_CO_ (%pred)	73.5 ± 23.6
ISWD (m)	227 ± 166
Haemodynamics	
mRAP (mm Hg)	11 ± 7
mPAP (mm Hg)	39 ± 15
PAWP (mm Hg)	14.0 ± 7.0
CO (L/min)	5.9 ± 1.9
CI (L/min/m^2^)	3.2 ± 0.9
PVR (WU)	4.8 ± 3.8
PVRi (WU.m^2^)	8.4 ± 6.3
PA saturations (%)	78 ± 8
Anatomical defect (%)	
Right superior vein	66.7
Total right	4.4
Left superior vein	23.4
Right and left superior vein	4.4
Total left	1.1

Haemodynamics measured at right heart catheterization include mRAP, mPAP, PAWP, CO, CI, PVR, PVRi and PA.

CI, cardiac index; CO, cardiac output; DL_CO_, diffusing capacity for carbon monoxide; FEV_1_, forced expiratory volume in 1 s; FVC, forced vital capacity; ISWD, incremental shuttle walking test distance; mPAP, mean PA pressure; mRAP, mean right atrial pressure; PA, pulmonary arterial; PAWP, PA wedge pressure; PVR, pulmonary vascular resistance; PVRi, PVR indexed for body surface area; WHO, World Health Organization; WU, Wood unit.

### Pulmonary venous anatomy

**Figure 2:**
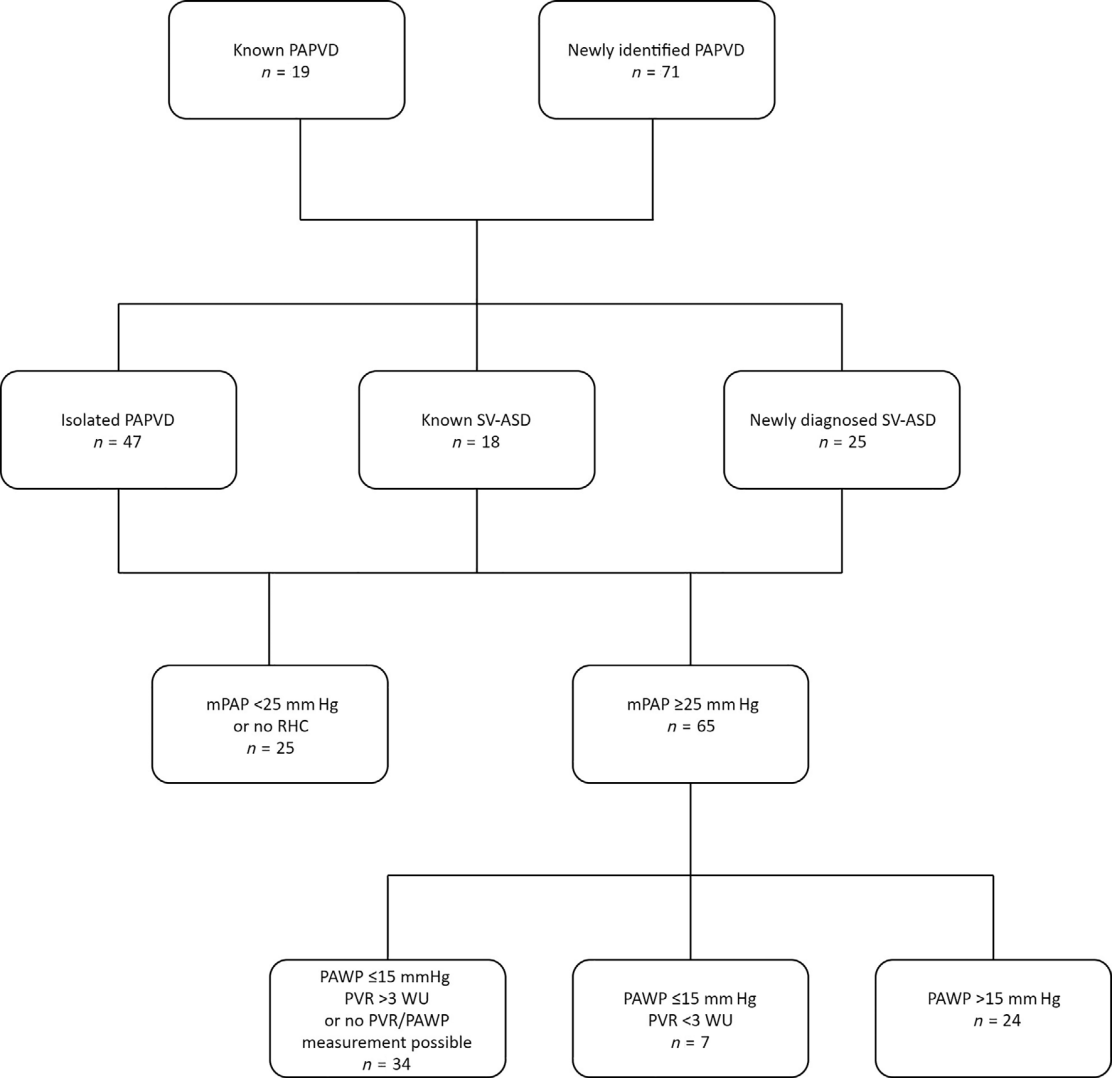
Flow chart demonstrating frequency of SV‐ASD and haemodynamic parameters in patients with PAPVD. mPAP, mean PA pressure; PA, pulmonary arterial; PAPVD, partial anomalous pulmonary venous drainage; PAWP, PA wedge pressure; PVR, pulmonary vascular resistance; RHC, right heart catheterization; SV‐ASD, sinus venosus atrial septal defect.

Seventy‐one patients (79%) were newly diagnosed with PAPVD following review at the unit; 49 of these 71 patients (69%) had previously undergone contrast‐enhanced CT locally where the anomalous venous drainage had not been appreciated. An SV‐ASD was visible on cross‐sectional imaging in 31 patients (34%); the SV‐ASD had not been previously diagnosed in 25 (81%) of these patients (Fig. 2). PAPVD was isolated (i.e. there was no evidence of an associated ASD) in 47 patients. Six further patients had previously undergone ASD repair but still had PAPVD.

The majority of patients had abnormal drainage limited to the right superior vein (*n* = 60, 67%; Table 1). Of these, 44 had isolated right upper lobe abnormal drainage while 16 had combined right upper and middle lobe abnormalities. Twenty‐one patients (23%) had an isolated left superior vein anomaly. Five patients (6%) had anomalous connection of an entire lung; the right lung was affected in four patients and the left lung in one patient.

### Pulmonary haemodynamics

Eighty patients (89%) underwent right heart catheterization (RHC) and 64 (79% of those undergoing RHC) were found to have PH as defined at the time of study enrolment by a mPAP ≥ 25 mm Hg. A further patient with Eisenmenger physiology did not undergo RHC. PH was post‐capillary (as defined by a PA wedge pressure (PAWP) > 15 mm Hg) in 24 (37%) patients.

Measurement of pressure in the wedged position of the pulmonary artery supplying lung with anomalous venous drainage will actually measure right atrial pressure. As the lobe in which the PAWP was measured was not recorded at the time of catheterization, we therefore compared the left atrial (LA) area, measured on CT, of those with a PAWP of ≤15 and >15 mm Hg. LA area was significantly larger (31.4 vs 20.1 cm^2^, *P* < 0.0005) in patients with a PAWP > 15 mm Hg.

### Cardiac anatomy

Cardiac chamber measurements for patients who had isolated PAPVD compared with those with an associated ASD are displayed in Table 2. Patients with isolated PAPVD had a smaller RV area (31.6 vs 38.6 cm^2^, *P* = 0.001). There was no significant difference for LA area (22.8 vs 22.9 cm^2^) or right atrial area (30.8 vs 33.0 cm^2^, respectively) between the two groups. Patients with an associated ASD did, however, have a larger pulmonary artery diameter (3.9 vs 3.4 cm, *P* = 0.003). Right atrial, RV and LA areas were larger in patients with an elevated PAWP (all *P* < 0.01) while there was a trend towards a larger pulmonary artery in those with an elevated PAWP (*P* = 0.053).

**Table 2:** Comparison of patients with isolated PAPVD and PAPVD + ASD†

	Isolated PAPVD (*n* = 47)	PAPVD and ASD (*n* = 37)	*P‐*value
CT measurements			
Aorta (cm)	3.1 ± 0.5	3.0 ± 0.5	0.286
PA (cm)	3.4 ± 0.6	3.9 ± 0.7	0.003
LA (cm^2^)	22.8 ± 9.3	22.9 ± 7.0	0.956
RA (cm^2^)	30.8 ± 14.3	33.0 ± 12.6	0.495
RV (cm^2^)	31.6 ± 8.5	38.6 ± 8.5	0.001
Qp:Qs (cardiac MRI)‡	1.5 ± 0.4: 1	2.2 ± 0.9: 1	0.006
DL_CO_ (%pred)	67.4 ± 24.1	80.6 ± 20.7	0.015
Haemodynamics			
mRAP (mm Hg)	10.9 ± 6.4	10.1 ± 6.3	0.609
mPAP (mm Hg)	36.7 ± 15.5	41.4 ± 15.7	0.209
PAWP (mm Hg)	13.2 ± 6.5	13.1 ± 7.1	0.955
CO (L/min)	5.9 ± 2.0	5.9 ± 1.7	0.962
CI (L/min/m^2^)	3.2 ± 1.0	3.3 ± 0.8	0.559
PVR (WU)	4.8 ± 3.9	4.7 ± 3.2	0.868
PVRi (WU.m^2^)	8.9 ± 7.4	8.5 ± 5.0	0.791
PA saturations (%)	76 ± 8	80 ± 7	0.064

Haemodynamics measured at right heart catheterization include mRAP, mPAP, PAWP, CO, CI, PVR, PVRi and PA.

† Patients with a previously closed ASD were not included in this analysis.

‡ Forty‐three patients had data available from cardiac MRI.

ASD, atrial septal defect; CI, cardiac index; CO, cardiac output; CT, computed tomography; DL_CO_, diffusing capacity for carbon monoxide; LA, left atrial; mPAP, mean PA pressure; mRAP, mean right atrial pressure; PA, pulmonary arterial; PAPVD, partial anomalous pulmonary venous drainage; PAWP, PA wedge pressure; PVR, pulmonary vascular resistance; PVRi, PVR indexed for body surface area; Qp:Qs, pulmonary to systemic shunt assessment; RA, right atrium; RV, right ventricular; WU, Wood unit.

### Shunt data

Qp:Qs as assessed by flow measurements at cardiac MRI (CMR, performed in 43 patients) was significantly higher in patients with an associated SV‐ASD compared to those without (2.2:1 vs 1.5:1, *P* = 0.006). Percent‐predicted DL_CO_ was significantly higher in patients with an associated SV‐ASD (81% vs 67%, *P* < 0.05). There was no significant difference in CMR‐derived Qp:Qs between those patients with a normal versus an elevated PAWP.

### Single versus multiple APVD

In those with isolated PAPVD, both Qp:Qs measured at CMR and DL_CO_ %pred were higher (1.8:1 vs 1.3:1 and 83% vs 63%, respectively, both *P* < 0.05) in those with anomalous pulmonary veins draining >1 lobe. An mPAP ≥ 25 mm Hg was observed in 27 patients with isolated anomalous drainage of a single pulmonary vein. Of these patients, 17 (63%) had a pulmonary vascular resistance (PVR) > 3 Wood unit (WU).

### Comorbidities

Additional possible causes of PH were present in 37 of 65 patients with PH (57%): elevated LA pressure (*n* = 18), significant lung disease (*n* = 8), combined elevated LA pressure and lung disease (*n* = 4), chronic thromboembolic disease (*n* = 4), cirrhosis (*n* = 2) and hereditary haemorrhagic telangiectasia (*n* = 1). One patient with chronic thromboembolic disease and one patient with cirrhosis also had an elevated PAWP. No additional causes of PH were identified in 28 patients (43%). Of these 28 patients, 17 (26% of those patients with PH) had a PVR > 3 WU. Seven (11% of those with PH) of these patients had isolated PAPVD, five of whom (8% of those patients with PH) had anomalous drainage of a single pulmonary vein.

### Follow‐up and outcomes

By the census date, 16 patients (18%) had died. Fifteen patients were already known to congenital heart disease units prior to being seen at our centre. Fifty‐three patients were subsequently referred by us to regional ACHD centres for assessment for re‐routing of anomalous pulmonary veins and/or closure of ASD. Fifteen patients were identified as being appropriate for surgical intervention (mPAP: 28 ± 8 mm Hg, mean PAWP: 11 ± 4 mm Hg, PVR: 2.6 ± 0.9 WU) and 13 chose to have surgical correction performed. Of these 15 patients, 11 had an associated SV‐ASD with 3 of the 4 patients without an SV‐ASD having abnormal drainage of >1 pulmonary vein. Twenty‐nine PH patients (mPAP: 50 ± 9 mm Hg, mean PAWP: 10 ± 4 mm Hg, PVR: 8.4 ± 3.5 WU) received a trial of PAH therapy (phosphodiesterase‐5 inhibitor, *n* = 19; endothelin receptor antagonist, *n* = 4; oral combination therapy, *n* = 2 and prostanoid‐based therapy, *n* = 4). Mean improvement in incremental shuttle walking distance from baseline to first follow‐up in the 25 patients with follow‐up data following initiation of PAH therapy was 23 m (*P* = 0.2).

## DISCUSSION

In the current study, we describe our experience of patients with PAPVD referred to a PH referral centre over a 10‐year period. To our knowledge, this represents the largest, to date, published cohort of patients with PAPVD‐associated PH.

**Figure 3:**
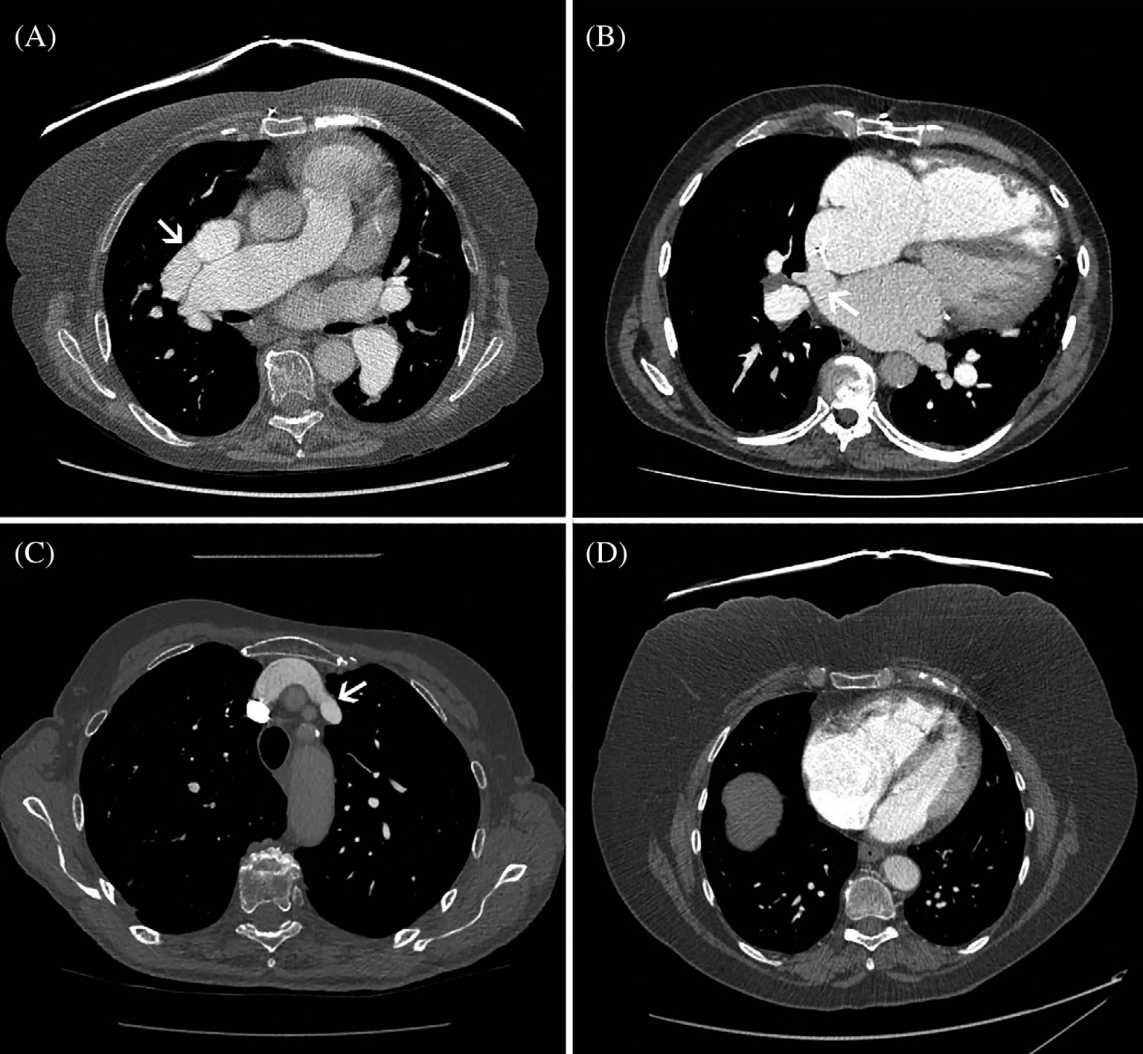
Contrast‐enhanced CT scans. (A) Anomalous drainage of right superior vein into SVC (arrow). (B) SV‐ASD (arrow). (C) Anomalous drainage of left superior vein into brachiocephalic vein (arrow). (D) Enlargement of anteriorly located right heart chambers in a patient with PAPVD. CT, computed tomography; PAPVD, partial anomalous pulmonary venous drainage; SV‐ASD, sinus venosus atrial septal defect; SVC, superior vena cava.

Of the 90 patients with PAPVD, 71 (79%) had not been previously identified as having congenital heart disease, despite CT scanning having been performed locally prior to referral in 49 (54%) patients. Furthermore, of the 31 patients with an ASD visible on CT, the diagnosis was newly made by our unit in 25. Jujo *et al*. previously studied eight patients identified with PAPVD at RHC over a 12‐year period and found that the diagnosis had been missed on initial reporting of CT scans in 50% of cases.^8^ Our new data highlight the importance of considering the possibility of APVD with or without ASD in all patients in whom PH is suspected. It can be difficult to identify APVD and SV‐ASD using standard transthoracic echocardiography.^9^ Many patients undergo CT scanning prior to referral to a PH specialist centre and, as part of a systematic evaluation of the thoracic CT, the course of all four pulmonary veins should be assessed (Fig. 3).

The majority of patients (67%) had anomalous drainage limited to the right superior pulmonary vein, with 29% involving left‐sided veins. Most previous clinical series of patients presenting with symptomatic PAPVD also reported predominantly right‐sided PAPVD, although two recent population‐based CT studies observed a left‐sided predominance.^1^, ^10^ The reason for this discrepancy is not clear. In keeping with previous studies, however, we identified that the majority of patients with PAPVD in our cohort were female.^1^, ^10^, ^11^

We hypothesized that patients with >1 anomalous vein in the absence of an ASD would have evidence of greater L‐R shunt than those with a single abnormal vein. Although there was no difference in terms of RV area on CT, we did observe a greater Qp:Qs and higher DL_CO_ %pred in patients with abnormal drainage of multiple lobes. This is consistent with previous observations by Majdalany *et al*. who reported 43 patients with isolated PAPVD seen during a 20‐year period; the vast majority of patients with RV dilatation who required surgery had >1 abnormal pulmonary vein.^12^ We similarly hypothesized that the 31 patients with associated ASD would have larger L‐R shunts than those without associated defects and, indeed, in those patients RV and pulmonary artery diameters and CMR‐assessed Qp:Qs were higher. We also hypothesized that this increased Qp:Qs would be reflected in standard non‐invasive physiological measurements. DL_CO_ %pred was higher in those patients with associated septal defects, in keeping with increased pulmonary capillary blood flow. These observations were contrary to those of Sahay *et al*. who did not see any effect of the presence or absence of an associated ASD difference on Qp:Qs in patients identified at a large ACHD centre over a 5‐year period.^13^ The number of patients in that study was, however, relatively small (*n* = 14, 6 of whom had PAH).

LA pressure as assessed by PAWP was elevated in 24 patients. In a normal person, each pulmonary vein drains approximately 25% of the total pulmonary blood flow.^14^ However, in anomalous pulmonary vein drainage, the shunt flow may be higher as the circulation is preferentially directed to the right side due to lower pressure in the RA and superior vena cava than in the left atrium. This effect becomes more pronounced in conditions that increase LA pressure such as systemic hypertension or left heart disease. It is interesting to note that the right‐sided chambers were significantly larger and there was a trend towards a larger pulmonary artery in those patients with an elevated PAWP.

In addition to elevated LA pressure, other potential causes of PH were present in 29% of patients with PH. In their report of 43 patients with isolated PAPVD, Majdalany *et al*. observed emphysema or interstitial lung disease in six patients, chronic thromboembolic disease in four patients and cirrhosis in two patients.^12^ It is possible that these associated conditions may act as a ‘second hit’ on a pulmonary vasculature already exposed to increased flow leading to the development of PH.

Optimal treatment of patients with PAPVD depends on the anatomical and haemodynamic picture. Our practice is to recommend subsequent referral of all patients with newly diagnosed PAPVD with or without ASD to their regional ACHD centre for an evaluation regarding surgical suitability. The role of PAH therapy in patients with PAH associated with PAPVD with or without ASD is not clear. Although there are several case studies and small case series reporting improvement with PAH therapy in patients with PAPVD and PAH, no such patients have been studied in randomized controlled trials.^2^, ^15^, ^16^, ^17^ Consideration for PAH therapy should therefore be done on a case‐by‐case basis taking into account pulmonary haemodynamics and the extent of L‐R shunt. In certain circumstances, reassessment of haemodynamics following PAH therapy may alter initial decisions regarding suitability for intervention.

This was a retrospective study and hence data for certain investigations, including inferior venocaval saturations enabling invasive Qp:Qs calculation, were not available for all patients. Some haemodynamic data provided should be interpreted with caution: the PA lobe in which the PAWP was measured was not recorded, and the PAWP will be inaccurate if the catheter is wedged in an artery supplying an anomalous lobe. In addition, previous studies have highlighted that cardiac output measured by thermodilution may be inaccurate in patients with LR intracardiac shunts, which may affect the data described in patients with PAPVD with associated ASD.^18^, ^19^ We have therefore presented data regarding Qp:Qs based on patients who had undergone Qp and Qs measurement at CMR.

In conclusion, undiagnosed PAPVD with or without ASD may be present in patients with suspected PH who are referred to a specialist referral centre. The presence or absence of PAPVD on cross‐sectional imaging should therefore be specifically assessed in all patients with suspected PH. Radiological and physiological markers of pulmonary blood flow are higher in patients with an associated SV‐ASD in keeping with increased L‐R shunt. Although many patients with PAPVD and PH may have other potential causes of PH, a proportion of patients diagnosed with PAH have isolated APVD in the absence of other causative conditions.

## Author contributions

Conceptualization: R.A.L., R.C. Data curation: R.A.L., C.G.B., A.B., S.B., A.C., P.C., C.A.E., K.E., N.H., C.H., J.H., P.J.J., C.J., S.M., J.O., V.P., S.R., I.S., A.J.S., A.A.R.T., D.G.K., R.C. Formal analysis: R.A.L., R.C. Investigation: R.A.L., C.G.B., A.B., S.B., A.C., P.C., C.A.E., K.E., N.H., C.H., J.H., P.J.J., C.J., S.M., J.O., V.P.,S.R., I.S.,A.J.S., A.A.R.T., D.G.K., R.C. Methodology: R.A.L. Project administration: R.A.L. Writing—original draft: R.A.L., S.R., I.S., R.C. Writing—review and editing: R.A.L., C.G.B., A.B., S.B., A.C., P.C., C.A.E., K.E., N.H., C.H., J.H., P.J.J., C.J., S.M., J.O., V.P., A.J.S., A.A.R.T., D.G.K., R.C.

**Abbreviations**
ACHD: adult congenital heart disease
APVD: anomalous pulmonary venous drainage
ASD: atrial septal defect
ASPIRE: Assessing the Spectrum of Pulmonary hypertension Identified at a REferral centre
CI: cardiac index
CMR: cardiac magnetic resonance
CO: cardiac output
CT: computed tomography
DL_CO_
: diffusing capacity for carbon monoxide
FEV_1_
: forced expiratory volume in 1 s
FVC: forced vital capacity
LA: left atrial
L‐R: left‐to‐right
mPAP: mean PA pressure
mRAP: mean right atrial pressure
MRI: magnetic resonance imaging
PA: pulmonary arterial
PAH: PA hypertension
PAPVD: partial APVD
PAWP: PA wedge pressure
PH: pulmonary hypertension
PVR: pulmonary vascular resistance
PVRi: PVR indexed for body surface area
Qp:Qs: pulmonary to systemic shunt assessment
RHC: right heart catheterization
RV: right ventricular
SV‐ASD: sinus venosus ASD
WU: Wood unit
